# Balance Improvement Effects of Biofeedback Systems with State-of-the-Art Wearable Sensors: A Systematic Review

**DOI:** 10.3390/s16040434

**Published:** 2016-03-25

**Authors:** Christina Zong-Hao Ma, Duo Wai-Chi Wong, Wing Kai Lam, Anson Hong-Ping Wan, Winson Chiu-Chun Lee

**Affiliations:** 1 Interdisciplinary Division of Biomedical Engineering, The Hong Kong Polytechnic University, Hong Kong, China; christina.ma@connect.polyu.hk (C.Z.-H.M.); duo.wong@polyu.edu.hk (D.W.-C.W.); ansonhomehk@hotmail.com (A.H.-P.W.); 2 Rehabilitation Engineering Research Institute, China Rehabilitation Research Center, Beijing 100068, China; 3 Li Ning Sports Science Research Center, Beijing 101111, China; gilbertlam@li-ning.com.cn; 4 Institute of Active Ageing, The Hong Kong Polytechnic University, Hong Kong, China

**Keywords:** falls, wearable sensors, balance, inertial motion sensors, force sensors, real-time biofeedback, sensory augmentation

## Abstract

Falls and fall-induced injuries are major global public health problems. Balance and gait disorders have been the second leading cause of falls. Inertial motion sensors and force sensors have been widely used to monitor both static and dynamic balance performance. Based on the detected performance, instant visual, auditory, electrotactile and vibrotactile biofeedback could be provided to augment the somatosensory input and enhance balance control. This review aims to synthesize the research examining the effect of biofeedback systems, with wearable inertial motion sensors and force sensors, on balance performance. Randomized and non-randomized clinical trials were included in this review. All studies were evaluated based on the methodological quality. Sample characteristics, device design and study characteristics were summarized. Most previous studies suggested that biofeedback devices were effective in enhancing static and dynamic balance in healthy young and older adults, and patients with balance and gait disorders. Attention should be paid to the choice of appropriate types of sensors and biofeedback for different intended purposes. Maximizing the computing capacity of the micro-processer, while minimizing the size of the electronic components, appears to be the future direction of optimizing the devices. Wearable balance-improving devices have their potential of serving as balance aids in daily life, which can be used indoors and outdoors.

## 1. Introduction

Falls and fall-induced injuries are major global public health problems [1,2,3]. Approximately 30% of people aged 65 or older living in the community and more than 50% of those living in residential care facilities or nursing homes experience falls every year [3,4,5,6,7]. The burden of falls and fall-induced injuries is heavy, as they can result in significant mortality and mobility [4,8], reduction of life span [9,10], reduced quality of life [10,11,12], and enormous health care costs [10,13]. Balance and gait disorders have been suggested to be the second leading cause of falls, just coming after accidents [14]. Multiple factors contributed to balance and gait disorders, including aging, sensory abnormalities, musculoskeletal disorders, neurologic disorders, cardiovascular diseases, infectious and metabolic diseases, and psychiatric conditions [15].

Since poor balance during standing and walking is the leading cause of falls, some researchers have used state-of-the-art technology to monitor balance performance and provide instant feedback reminding on the necessary postural adjustments in an attempt to improve balance. Floor-mounted force plates [16,17,18,19] and motion capture systems (with infra-red cameras detecting positions of reflective markers attached to human body) [20,21,22] allowed tracking of real-time position of center of pressure (COP) and center of mass (COM) of human body, respectively. When the position of the COP/COM exceeded a pre-defined range, or the so-called dead zone, some instant reminding feedback would be delivered to the users, which were shown to be able to reduce the user’s postural sway [16,17,18,19,20,21,22]. Although the above-mentioned systems could monitor the balance performance accurately and reliably, the non-portable design limited their applications to in-door use only. This required patients to go to hospitals/laboratories to receive training constantly, which led to low level of continuity and adherence of training [23,24]. This also impeded their use in real-world environments [25].

To make the overall system portable, some other devices using wearable sensors to monitor the balance performance were developed [26,27,28,29,30,31,32]. This trend has been growing as it allowed assessment of postural sway by built-in portable sensors, and monitoring the type, quality, and quantity of daily activities of the users outside laboratory environment [25]. Various types of wearable sensors, including inertial motion sensors [33,34,35,36,37,38,39,40,41,42,43,44,45] and force sensors [28,29,46,47,48,49,50,51], have been widely used to detect body sway and work as a real-time balance performance monitoring device. Inertial motion sensors (accelerometers, gyroscopes and magnetometers) were mounted on user’s trunk, head, or lower limbs to capture torso, head or other body segments’ movements to determine any tilts in mediolateral and anteroposterior directions [33,34,35,36,37,38,39,40,41,42,43,44,45]; and thin-film force/pressure sensors were put at the plantar surface of foot to measure the ground reaction force information [28,29,46,52,53,54,55]. The advantages of wearable sensors lied in the fact that they allowed the balance monitoring to be conducted anywhere and anytime, which provided them the potential to be used as balance monitoring aids in daily life. It has also been suggested that the low-cost and portable sensors were accurate and reliable enough, and might be able to replace the conventional clinical instruments used for assessments [56].

All those wearable inertial motion and force sensors were connected to computers/smartphones, which analyzed postures by interpreting the body motion and plantar force signals. To provide biofeedback, control signals were then sent to a display (visual feedback) [31,57,58,59], an audio device (audio feedback) [60,61,62], some electrodes (electrotactile feedback) [63], or some vibrators (vibrotactile feedback) [28,29,34,37,39,40,41], based on the measurement of the sensors. Excessive postural sway during standing and walking [28,29,47], and high level of gait variability [48,49] had been considered to be indicators of poor balance. When the user’s balance detected by the wearable sensors was poor, reminding feedbacks were produced. Those devices have been applied successfully in healthy young [28,45,48] and older adults [28,29,64,65], patients with stroke [47,49,50], spinal cord injury [63], diabetic neuropathy [43,66], Parkinson’s disease [42,47,67], vestibular loss [42,68], multiple sclerosis [42], and lower-limb amputees [51]. The wearable characteristic allowed the coaching and balance training to be conducted at home and any other places. High satisfactory level of accuracy, usability, and safety of these devices was achieved [44,45,48,69].

There are a growing number of wearable devices aimed at improving static and dynamic balance in patients or aged population, and several relevant reviews have been conducted. Razak *et al.* reviewed recent wearable foot plantar pressure measurement systems [70]. They mainly evaluated the accuracy and reliability of force sensors on measuring pressure distribution under the foot, without considering the effect of the provided corresponding biofeedback on balance. Baram performed a review of the literature to examine the effects of body motion sensors and sensory feedback on gait improvement in patients with neurological disorders [71], and concluded that patients with Parkinson’s disease, multiple sclerosis, stroke, and cerebral palsy could improve their balance and gait upon using these devices. Nevertheless, this review included a number of non-wearable sensors and the biofeedback information was only limited to visual feedback, which might limit their use to indoor hospital training only. Recently, Habib *et al.* reviewed the effects of smartphones on detecting falling risks and preventing falls [72]. They concluded that smartphones were user friendly and thus enhanced the usability/compliance of the biofeedback systems. However, their review did not consider the potential of other wearable sensors such as force sensors. Horak *et al.* summarized the advantages of using body-worn movement monitoring technology for balance and gait rehabilitation [42]. Based on the measurements of body-worn movement monitors, clinicians could monitor the compliance of home-based rehabilitation training and identify the recovering process of their patients. However, they mainly concentrated on the monitoring function of wearable sensors, without thoroughly looking into the effects of additional real-time biofeedback, on patients.

This paper extends previous efforts by reviewing various biofeedback systems with plantar force and inertial motion sensors that are wearable. A better understanding of the effective design of previous devices can shed new lights on future design of wearable biofeedback devices to improve balance and reduce falls. The objectives of this study were: (1) to review the underlying wearable sensing mechanisms and commonly employed balance assessment methods; (2) to summarize the key design concepts of biofeedback systems using wearable motion sensors; (3) to examine the effectiveness of biofeedback systems with wearable sensors on improving balance performance; and (4) to suggest future design directions of wearable biofeedback systems that could potentially make them sustainably viable.

## 2. Wearable Sensing Mechanisms

Inertial motion and plantar force sensors are the most commonly used sensors to objectively evaluate body motion. Their small size allows them to be wearable. Inertial motion sensors can detect the postural sway by measuring the linear acceleration, angular velocity, and direction of body movements. Plantar force sensors can detect the postural sway and gait variability by measuring the COP trajectory at plantar surface of foot and stance/swing time during walking, respectively. The range, average, and standard deviation of these quantities are used to evaluate the degrees of postural sway and gait variability. Generally, increases in postural sway and gait variability are interpreted as a deterioration of balance performance [26,27,73]. An overview of these wearable sensors is summarized in Table 1, including the type and location of sensor, and outcome measurement. More detailed descriptions of sensing mechanism of each sensor are summarized in the following texts.

### 2.1. Inertial Motion Sensors

State-of-the-art inertial measurement units (IMU), based on microelectromechanical systems (MEMS), can incorporate up to all nine axes of sensing in a single integrated circuit package. The units consist of a tri-axial accelerometer, a tri-axial gyroscope, and a tri-axial magnetometer [69,74], measuring linear acceleration, angular velocity, and direction, respectively. Such information could be further processed to reveal the orientation/inclination of human body or body segments. Inertial motion sensors were able to identify increased trunk and head inclination [36,75], and decreased coordination among lower-limb joints [43], which were interpreted as poorer balance performance. The detailed sensing mechanisms of each sensor and the working mechanism after incorporating them together are described below.

#### 2.1.1. Accelerometers

The tri-axial accelerometers could detect the acceleration of X, Y, and Z movements in a three dimensional space. The underlying mechanisms are that the accelerometers independently measure the respective acceleration in each of the three directions, or the so-called “g-force”, as a vector quantity. The output of an accelerometer is normally expressed as in Equation (1):

(1) $$\vec{a}=\vec{g}+\vec{a_{l}}+\vec{\varepsilon }$$

where $\vec{a}$ is the output of an accelerometer, $\vec{g}$ is the gravity acceleration, $\vec{a_{l}}$ is the linear acceleration, and $\vec{\varepsilon }$ is the noise in sensor coordinate frame [76].

Based on the detected changes of magnitude and direction of g-force, the direction of linear movements of an object could be obtained [77]. This is how the microelectromechanical accelerometers mounted on body segments detect the direction of movement of various body segments.

#### 2.1.2. Gyroscopes

The gyroscopes could measure the extent and rate of rotation in a three dimensional space (roll, pitch, and yaw) [77]. They are designed based on the theory of Coriolis effect, which states that in a frame of reference rotating at angular velocity, a mass moving with velocity experiences a force as shown in Equation (2) [77]:

(2) $$\vec{F_{c}}=-2m\left(\vec{\omega }\times \vec{\vartheta }\right)$$

where $\vec{\omega }$ represents the angular velocity, *m* represents the mass, $\vec{\vartheta }$ represents the velocity, and $\vec{F_{c}}$ represents the experienced force [77].

The gyroscope involves a spinning disc in which the axis of rotation is free to assume any orientation. The orientation of this axis is not affected by instantaneous tilting or rotation of the mounting object according to the conservation of angular momentum, which allows the gyroscopes to detect more accurate movement within a three-dimensional space than using accelerometers only in a relative short time period [78].

#### 2.1.3. Magnetometers

The magnetometers could provide the direction information or the absolute angular measurements relative to the Earth’s magnetic field [76]. The detected vector components of a magnetic field consist of declination (the angle between the horizontal component of the field vector and magnetic north) and inclination (the angle between the field vector and the horizontal surface).

#### 2.1.4. Integrated Sensing Mechanism

Either a tri-axial accelerometer or a tri-axial gyroscope can already provide orientation information of an object. However, the accelerometer only measures linear acceleration along one or several axes, and the signal measured by an accelerometer is biased by gravity [77]. It also has high level of signal noise at the onset of acceleration [76,78]. The gyroscope measures instantaneous angular velocity accurately, but additional error will accumulate over a period of time or even seconds when it is not undergoing any rotations [77], leading to inaccurate measurement of pitch/roll angle in a relative long time period [76]. Thus, to achieve accurate and sufficient measurements of orientation in both short and long time periods, both accelerometers and gyroscopes are needed which calibrate each other [78]. However, the combined use of accelerometers and gyroscopes could only provide orientation information of body motion in a three dimensional space, without providing the absolute direction information relative to the Earth’s magnetic field [77]. An additional magnetometer measuring direction information is needed to help monitor the body segment’s motion more clear, as a universal reference is added thereafter [76].

### 2.2. Plantar Force Sensors

In addition to the inertial motion sensors that measure the body inclination directly, the force sensors located at plantar surface of foot measured the plantar force/pressure information, which could be further analyzed to assess balance performance. Plantar foot surface is 3-dimensional, with the presence of foot arches. The degrees of freedom of these plantar pressure measurements are determined by the number and positions of the sensors being used. The common balance indicators derived from plantar force sensors include COP trajectory and variability of spatial-temporal gait parameters. The force sensor’s location at insoles and the force measured by multiple force sensors could be used to calculate the location and trajectory of center-of-pressure (COP) (Equation (3)):

(3) $$COP_{x}=\frac{\int \left[x\times p\left(x\right)\right]dx}{\int \left[p\left(x\right)\right]dx}\;\text{and}\;COP_{y}=\frac{\int \left[y\times p\left(y\right)\right]dy}{\int \left[p\left(y\right)\right]dy}$$

where *P* is the pressure at plantar surface of each foot, *p(x)*, *p(y)* is the pressure depends on the distance *x*, *y* from a reference line, ∫[]*dx*, ∫[]*dy* is the integration of a continuous function.

The parameters calculated based on the trajectory of COP during standing [79] and walking [80], including mean velocity and range, could be used to evaluate postural stability. Maintenance of good postural stability is a key opposite indicator of fall risks [81]. Increased medial-lateral (ML) displacements of COP is generally interpreted as an overall deterioration of postural stability during standing and walking [73]. In addition, the COP variability during walking is associated with dynamic balance performance, greater COP variability generally indicated poorer balance [82]. The parameter commonly used to assess COP variability during walking is the root-mean-square deviation of step-by-step COP [82].

In addition to the COP variability, gait variability could also be determined based on the variation of stance and swing time, and weight-bearing asymmetry measured by the force sensors put under the left and right feet [51]. Some systems measured the timing of force applications at the plantar surface of the heel and the forefoot to calculate the stance and swing time. Symmetry of stance and swing time, and weight-bearing between two legs was used as indicators of dynamic balance performance during walking [51].

## 3. Evaluation of Static and Dynamic Balance

To evaluate the clinical values of biofeedback systems with wearable sensors, balance assessments have to be conducted comparing the differences between with and without the use of the systems.

Assessment of balance control is important to evaluate risk of falling and can be grouped into two categories: static and dynamic [83,84]. Static balance control task requires the subjects to establish a stable base of support and try to maintain the COP within this base of support during the assessment. Sometimes, some challenges, such as standing with eyes-closed or standing on a perturbation floor, were added to increase the difficulty of maintaining balance. However, daily life involves a lot of movements which suggests static balance control task alone may not be enough. In dynamic balance control tasks, subjects are asked to do some degrees of body movements, such as rising from a chair and turn 360°, without compromising the established base of support, which more closely mimic the demands of physical activities than the static postural control tasks [84].

Instrumented and non-instrumented tests have been adopted to evaluate static and dynamic balance. Instrumented tests commonly measure the movement of COP during standing using a floor-mounted force plate, or movement of COM during standing/walking using inertial motion sensors integrated in a smartphone or infrared cameras and reflective markers adhering to the body bony landmarks. Non-instrumented tests mainly consist of some clinical tests, balance assessing scales and questionnaires. They are widely applied in clinical practice, as these tests do not rely on bulky electronic instruments and are easy to administer. Only simple objects, e.g., stopwatch, pencil, and paper, are used to do the recording. Sometimes, both instrumented and non-instrumented approaches are used to obtain a better picture of balance performance.

### 3.1. Instrumented Tests

#### 3.1.1. Measurement of Center of Pressure (COP) Displacement—Static and Dynamic Balance Assessments

While maintaining the COP within the base of support is the key to maintain balance [85], balance test has been conducted to evaluate the tiny movements of COP during standing and walking [81]. COP is defined as the position of the global ground reaction force vector that adapts to the body sway and position [79]. It represents a weighted average of all the pressure over the surface of area in contact with the ground [86]. Parameters derived from the COP signal provided objective information on postural control mechanism, which can be used to detect balance deficiency, predict falling risk and evaluate the efficacy of balance training programs or interventions [87]. Larger COP-based outcome measure is typically described as a deterioration of postural stability [88]. While the force plate-captured COP movements could indicate static balance control during standing, some in-shoe plantar force measurement systems incorporating numerous force sensors at plantar surface of each foot could monitor the COP trajectory during walking and other functional tasks to evaluate the dynamic balance control, as described in previous sections [26,27].

#### 3.1.2. Measurement of Center of Mass (COM) Displacement—Static and Dynamic Balance Assessments

The COM is defined as the point that equivalent of the total body mass in a global reference system and is the weighted average of the COM of each body segment in a three dimensional space [86]. Measurement of COM displacements using cameras and reflective markers adhered to the body bony landmarks [63,67], or the inertial motion sensors attached to the posterior trunk near the COM (e.g., midpoint of posterior superior iliac spine) [36,43,89] are common methods to assess postural balance. The estimation of COM requires an accurate anthropometric model, which composes of several body segments such as head, trunk, upper limbs and lower limbs; as well as a full kinematic description of each marker that attached to the distal and proximal bony landmarks of body segments [90]. The reflective markers are generally attached to the lateral side of joints to facilitate camera capturing. Common bony landmarks used to adhere the reflective markers consist of acromion, anterior superior iliac spine (ASIS), posterior superior iliac spine (PSIS), knee joint center, lateral malleolus, suprasternal, styloid process, tip of 2nd toe, greater trochanter, and xyphiod [90]. With the sufficient anthropometric model, the location of COM could be calculated as in Equation (4):

(4) $$COM=\frac{1}{N}\overset{n}{\underset{i=1}{\sum }}COM_{i}\times m_{i}$$

where *M* is the total body mass, *m_i_* is the mass of the *i*th segment, *COM_i_* is the coordinate of the *i*th segment, and *N* is the number of segments defining the body COM [90]. Generally, the inverted pendulum model is adopted to evaluate the static postural stability, which required the vertical projection of COM on the ground to be within the area of base of support in static situation [91].

Some researchers also proposed the use of extrapolated COM (XcoM), based on the inverted pendulum theory, to evaluate the postural stability in dynamic situations. The extrapolated center of mass is defined as the center of mass position plus the center of mass velocity multiplied by a parameter related to the subject's leg length. The XcoM usually moves away from the COP, and the COM ultimately follows the XcoM. To maintain balance, the position (the vertical projection) of the COM plus its velocity times a factor $\sqrt{l/g}$ should be within the base of support, where *l* being leg length and *g* the acceleration of gravity [91,92]. The position of XcoM is calculated as in Equation (5):

(5) $$XcoM=x+\frac{v_{x}}{\omega_{0}}$$

where *XcoM* is the extrapolated center of mass, *x* is the projection of the COM position on the ground, *v_x_* is the velocity of COM, $\omega_{0}=\sqrt{g/l}$ is the Eigen-frequency of the inverted pendulum, *l* is the leg length, and *g* is the acceleration of gravity [91,92]. Larger COM- and XcoM- based outcome measures indicate poorer balance control in static and dynamic situations, respectively [91,92].

#### 3.1.3. Balance Perturbations

In addition to conventional static and dynamic balance assessments, the balance control would be more challenging by adding some perturbations (e.g., cognitive demanding tasks or unstable support surfaces/devices/environments). The common interventions include requiring the subjects to stand/walk on a foam pad [93,94] or moving support surface [95], be pushed/pulled suddenly during quite standing with eyes-closed [96], or perform some dual tasks while trying to maintain balance. The postural sway is usually evaluated by measuring the displacement of COP or COM [93,94].

### 3.2. Non-Instrumented Tests

#### 3.2.1. The Romberg Test—Static Balance Assessment

Common static balance tests include the Romberg test, which compares postural stability between the eyes-open and eyes-closed states during standing with feet together, upper limbs crossed over and hands rested on the opposite shoulders [97]. It evaluates the functions of the lower-limb proprioceptive spinal reflex arcs by placing the subjects in a more challenging postural position and only allowing the use of proprioceptive and vestibular inputs to maintain upright position. Patients with proprioceptive impairments could stand stably and comfortably with eyes open, but would reveal a significantly increased postural sway, stumble, or even fall when eyes are closed [98]. The clinicians visually observe the degrees of postural sway in eyes-open and eyes-closed conditions. Significantly increased postural sway in eyes-closed condition than in eyes-open condition or even unable to maintain this posture would suggest that the patients have balance deficits.

#### 3.2.2. Tandem Standing—Static Balance Assessment

The so-called Tandem Standing Test is the “sharpened” or “challenging” Romberg test and asks the subjects to stand in the tandem position, which is to stand with the heel of front foot touching the toe of back foot. In this standing condition, the proprioceptive input from the ankle joints would become more discordant compared with the vestibular and visual inputs, which makes the test more sensitive to dysfunctions of proprioceptive sensory systems [99].

#### 3.2.3. Limits of Stability (LOS) Balance Test—Static Balance Assessment

Evaluation of LOS requires the subjects to stand quietly first, then lean their trunk forward as far as possible while maintaining the maximum leaning position without loss of balance [100]. The subject’s maximum forward leaning distance is measured to evaluate balance control. A longer leaning distance represents better static balance control [100]. An additional force platform could be used to measure the range of COP excursion when conducting this test [100].

#### 3.2.4. The Star Excursion Balance Test (SEBT)—Dynamic Balance Assessment

The SEBT has been a widely adopted dynamic balance test to assess presence of pathological conditions and effectiveness of interventions, with high level of reliability and validity to identify dynamic balance deficits [84]. It requires the subjects to stand with one foot fixed at a point, and the other leg (non-stance) to reach maximally to touch the points along eight designated lines/directions that are 45 degrees to each other on the ground [101]. The averaged values of the maximum reached distance in eight designated lines of the subjects are used as an index of dynamic postural control. A longer reached distance indicates better dynamic postural control [84]. With appropriate instructions and normalization of the reaching distances, these assessments can be compared between pre- and post-interventions to quantify changes in postural control [84].

#### 3.2.5. The Tandem Gait Performance—Dynamic Balance Assessment

The tandem gait is the walking pattern with the heel of front foot touching the toe of back foot at each walking step [22,102]. Generally, subjects are required to walk 10 steps when performing the tandem walking. The degree of postural sway and ability of performing this test could be visually observed by the clinicians and researchers. Excessive postural sway or even unable to perform this test determines the subjects as having poor balance control. The spatial-temporal gait parameters and displacement of COM could also be captured using cameras or inertial motion sensors to evaluate balance control [22].

#### 3.2.6. The Berg Balance Scale (BBS)—Dynamic Balance Assessment

The BBS is a balance assessment questionnaire that measures subjective perceived balance ability while performing each of the 14 daily activities, including transferring, standing unsupported, rising from a sitting to standing position, tandem standing, turning 360° and single-leg standing [103,104]. The score is given based on the researcher’s/clinician’s perception of subject’s balance while performing the test [103,104].

#### 3.2.7. Timed Up and Go test (TUG)—Dynamic Balance Assessment

The Timed Up and Go test (TUG) is commonly employed to detect dynamic balance deficits in patients and elderly people [105]. When performing this test, the subjects are required to stand up from an armchair, walk a distance of 3 m, turn around, walk back to the chair, and sit down. The time of performing this test is measured by the stopwatch. A score of 1 to 5 based on the researcher’s/clinician’s perception of the subject’s risk of falling during the test is given [106].

## 4. Review on Previous Studies

### 4.1. Inclusion Criteria

#### 4.1.1. Types of Participants

This review considered studies that included healthy adults, as well as patients with balance disorders, including stroke, neuropathy, lower-limb amputation, vestibular diseases, cerebral palsy, spinal cord injury, and Parkinson’s disease. Studies were included in this review only if the participants were adults aged 18 years or over.

#### 4.1.2. Types of Sensors and Feedback

This review considered studies that used wearable sensors to detect balance and provided instant biofeedback based on the detected information. The sensors included wearable inertial motion sensors (accelerometers, gyroscopes, and magnetometers) and force sensors placed at plantar surface of foot. Studies that provided visual, auditory, electrotactile or vibrotactile biofeedback were included in this review.

#### 4.1.3. Types of Intervention Outcomes

This review considered studies that included the following intervention outcome measures: (1) instrumented measurements, including displacement of COP, COM, spatial-temporal and kinematic gait parameters; and (2) non-instrumented measurements, including standard clinical assessments, questionnaires, and verbal reports. Instrumented measurements were conducted using either the wearable sensors integrated in the biofeedback systems or extra sensors that were not part of the biofeedback system. Non-instrumented tests were conducted based on the examiner’s subjective observation, and the results of questionnaires and verbal reports.

#### 4.1.4. Types of Studies

This review considered experimental study designs, including randomized controlled trials, non-randomized controlled trials, quasi-experimental, before and after studies, prospective and retrospective cohort studies, case control studies, and analytical cross sectional studies for inclusion. This review also considered descriptive epidemiological study designs, including case series, individual case reports, and descriptive cross sectional studies for inclusion.

### 4.2. Searching Strategy

**Figure 1:**
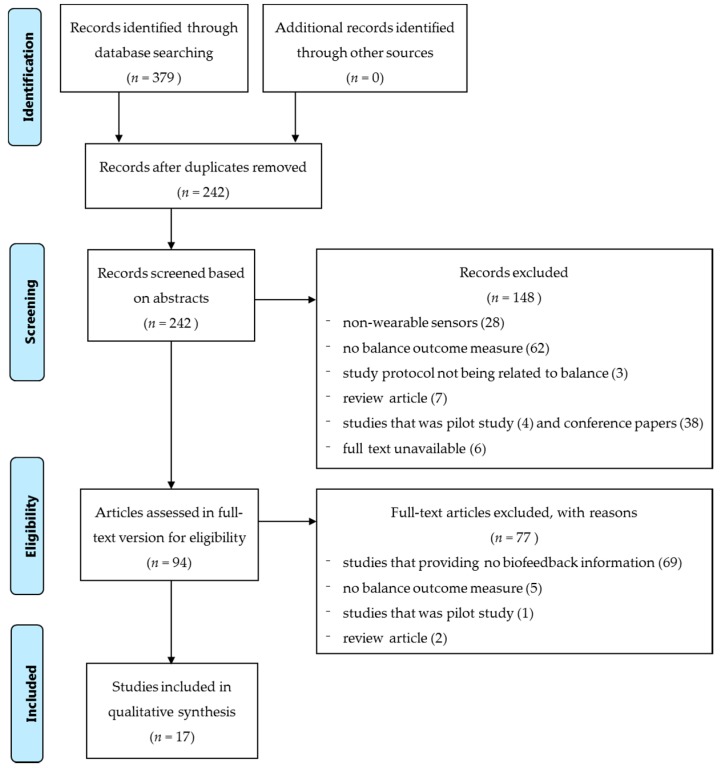
Flow chart of searching results and screening strategy.

Published studies were searched following the guidelines of the standardized critical appraisal instruments from the Joanna Briggs Institute Meta-Analysis of Statistics Assessment and Review Instrument (JBI-MAStARI). A three-step search strategy was employed. An initial search at MEDLINE only was undertaken to analyze the words contained in the titles and the abstracts, and the index terms that were used to describe the articles. The aim of this search was to identify appropriate keywords (Step 1). A second search was then undertaken to use all identified keywords and index terms to search across all included databases to identify all papers suitable for this review (Step 2). Thirdly, the reference lists of all identified reports and articles were also searched for more relevant studies (Step 3). Studies published in English and published from 1995 to 2015 were included in this review. The searching results are shown in the following flow chart, a total of 379 publications were found (Figure 1). The keywords that been identified in Step 1 and used for paper searching were: sensors; wearable sensors, force sensors, inertial motion sensors, accelerometer and gyroscope, sensory augmentation, sensory stimulation, biofeedback, balance, balance training, and postural stability. The databases that have been searched in Step 2 included: Web of Science, MEDLINE, and Google Scholar.

### 4.3. Assessment of Methodological Quality

Quantitative papers selected for retrieval were assessed for methodological validity prior to inclusion in the review, following the instructions of JBI-MAStARI. Two reviewers, who were not blinded to the authors and journals of the publications, independently assessed the quality of each included study in terms of grade of recommendation and level of evidence using the scoring protocol developed by the Oxford Centre for Evidence-based Medicine [107]. This scale includes ten levels of evidence divided into four levels of recommendation (Table 2 and Table 3).

The highest level of evidence is systematic review (with homogeneity) of randomized controlled trials, and the lowest is expert opinion without explicit critical appraisal. A Downs and Black quality list was adopted to assess the reporting quality, methodological design, external validity and internal validity, and statistical power of all included randomized and non-randomized studies following the standard procedures as specified in [108] (Table 4). The scoring of most items in this checklist is based on the simple answers of “yes”, “no”, or “unable to determine”. Any disagreements of assessing results that arose between the two reviewers were resolved through discussion, or with a third reviewer.

### 4.4. Searching Results and Screening Strategy

Figure 1 shows the searching result and process of screening the publications. A total of 379 publications were identified after the 3-step literature search. After removing any duplicated publications, 242 publications remained. An initial screening of the abstracts removed a further 148 publications for not conforming to the inclusion criteria, which involved (1) non-wearable sensors (28 publications); (2) no balance outcome measures (62 publications); (3) study protocols not being related to balance (three publications); (4) review articles (seven publications); (5) studies that were pilot studies (four publications) and conference papers (38 publications), and (6) unavailable full text (six publications). The remaining 94 publications were further reviewed in their full-text versions to confirm they strictly met the inclusion criteria. A total of 77 publications were then excluded, as they: (1) did not provide sufficient biofeedback information (69 publications); (2) did not have any balance outcome measures (five publications); (3) were pilot studies (one publication); and (4) were review articles (two publications). Finally, seventeen publications met the inclusion criteria and were included in this review (Figure 1).

### 4.5. Data Collection and Data Synthesis

Qualitative data were extracted from papers and included in the review using the standardized data extraction tool from JBI-MAStARI. The data extracted included specific details about the interventions, populations, study methods, and outcomes of significance to the review question and specific objectives.

Studies were then summarized according to the following characteristics: methodological quality and level of evidence; study design; sample size; sample characteristics (age and gender); key characteristics of the device; follow-up time; outcome measures (for static and dynamic balance improvement); and results. The summary of these study characteristics were demonstrated using figures and tables in the following sections. Data synthesis using a meta-analysis was not possible due to the variety of study designs, methodologies, and outcome measures.

### 4.6. Methodological Quality and Level of Evidence

Seventeen publications were included in this review (Table 5). In terms of level of evidence and strength of recommendation [107,109], three publications were considered to be level 1B (individual randomized controlled trials with narrow confidence intervals), with recommendation of grade A (strong recommendation that is expected to be followed, unless there are compelling reasons to deviate from the recommendation in an individual) [43,47,50]. Fourteen were considered to be 3B (individual case-control study), with recommendation of grade B (weak recommendation that consideration should be given to follow the recommendation) [28,32,36,48,49,51,63,64,65,67,68,75,89,110]. These seventeen studies have some methodological weaknesses [32,36,43,47,48,49,50,51,63,64,65,66,67,68,75,89,110,111], including lack of randomization or double-blindness; only two of them incorporated blinded assessors [28,50]. The detailed assessing results of all included studies in terms of quality of reporting, internal validity (bias and confounding), external validity, and power using the Downs and Black Quality List are summarized in Table 6. Most of these studies revealed high level of reporting quality and internal validity. However, the quality of external validity and statistical power was relative low (Table 6). This review does not include any meta-analysis on the effective, as the included number of studies (with high level of evidence) is too small for that.

### 4.7. Sample Characteristics

As shown in Table 7, sample characteristics varied across studies. Sample size ranged from 1 [63] to 39 [43]. The subjects were predominantly males. The subjects recruited in the included studies consisted of healthy young and older adults, patients with diabetes, Parkinson’s disease, stroke, spinal cord injury, vestibular loss, and amputees. The inclusion and exclusion criteria regarding physical and cognitive functioning were different, as well as the clinical tests to evaluate these characteristics. Most studies did not specify the cognitive status; only three of them verified that the subjects did not have cognitive disorders with clinical assessments [36,43,50], and another two of them required the participants to be able to follow the experimental instructions [28,47] but did not state any relevant clinical assessments to verify the cognitive ability of subjects. Physical status were assessed by clinical tests, including Hoehn and Yahr Scale [47,67], Cafe 40-Functional Independence Scale [47], Modified Ashworth Scale [50], Air Force Class III equivalent physical examination [111], as well as self-reported independent walking abilities [36,43,47,50]. The history of falls was only specified in three publications [28,67,68]. The initial balance performance of subjects also varied across studies, though most studies involved subjects who encountered balance disorders (Table 7).

### 4.8. Types of Sensors, Biofeedback, and Balance Outcome Measurement Methods

Table 8 summarizes the types of wearable sensors and biofeedback adopted in the biofeedback devices. Generally, inertial motion sensors were used to measure the postural sway or lower-limb joint co-ordinations in medial-lateral and anterior-posterior directions during standing and walking [32,36,43,47,63,64,65,67,68,75,89,110]. Some studies put the inertial motion sensors at the lower back near the location of COM to assess postural sway [63,64,75,89,110]; at the shank, thigh and lower back in an attempt to estimate the lower-limb joint co-ordinations by measuring joint angles [43,47,66]; or at the head and trunk to measure the inclination of head and torso [32,65,67,68,111]. Most studies developed a new device consisted of various inertial motion sensors [36,43,47,66,67], while some studies directly used the smartphones equipped with inertial motion sensors to do the measurements [89].

Force sensors were attached to the plantar surface of foot to measure the ground-reaction-force information, which were processed and used to estimate the weight bearing asymmetry between affected and sound sides [47,49,50]; evaluate overall postural stability by measuring the plantar force distribution information [28]; or assess the step-to-step gait variability (speed, quality, symmetry, and stability) by detecting the gait phrases, mainly heel-strike and toe-off [35,48]. One study put only one plantar force sensor at heel to evaluate weight bearing symmetry between legs during standing [50], while some other studies put two or more force sensors at heel, metatarsal heads, and toes to detect postural sway during standing and gait phrase transitions [28,47,48,49,51].

Different kinds of biofeedback have been provided (Table 8). Single or multiple types of biofeedback information were provided, including visual [43,47,51,67,75,110], auditory [43,50,64,65,89], vibrotactile [28,32,36,48,49,68,110], and electrotactile [51,63,111]. The visual biofeedback information were usually shown on a large screen [43,51,67,75,110], or a smaller one, e.g., a tablet computer [47]. The auditory biofeedback was usually delivered through a headphone. Meanwhile, the electrotactile and vibrotactile feedbacks were usually provided through electrical or vibrating stimulators directly to the surface of skin, except one study adopted surgically implanted stimulators at the user’s trunk and lower-limb bilateral muscle groups [63].

The outcome measures of balance performance incorporated in these studies varied with respect to assessment specificity (Table 9). Of all the 17 studies that were reviewed, twelve assessed the static balance only [28,32,36,43,63,64,65,67,68,75,89,110], two assessed the dynamic balance during walking only [47,48], and the rest of them assessed both static and dynamic balance [49,50,51]. Most studies evaluated the immediate effects of these devices by comparing the balance ability between pre- and post- intervention. Only two studies assessed the long-term effect of the devices, which allowed the users to use the devices for three to six weeks [47,50]. Some of the studies, which recruited healthy subjects, required the subjects to stand with their eyes closed on a perturbation floor [28,32,64,89,111], or a soft foam surface [75,111].

Both instrumented tests and non-instrumented tests were used for balance evaluation. The studies used self-contained wearable sensors only [36,43,48,49,64,89], external assessing devices (e.g., force platform, motion capture system) only [28,50,51,63,65,67,68], or both of them to evaluate balance performance [32,47,75,110]. Of the studies incorporating instrumented tests, six assessed postural stability during standing using a floor-mounted force platform [28,32,65,68,75,111], four assessed postural control during standing and/or walking using a motion capture system [50,51,63,67], and ten assessed static and dynamic postural sway using the inertial motions sensors or force sensors self-contained in the fabricated biofeedback devices [36,43,47,48,49,64,75,89,110,111]. Of the studies incorporating non-instrumented tests, questionnaires and clinical tests, such as Berg Balance Scale and Timed Up and Go test, were used [47,48,50]. Generally, the non-instrumented tests were used as a secondary assessment of balance, in addition to the instrumented tests [47,48,50].

### 4.9. Summary on the Effectiveness of the Devices

**Figure 2:**
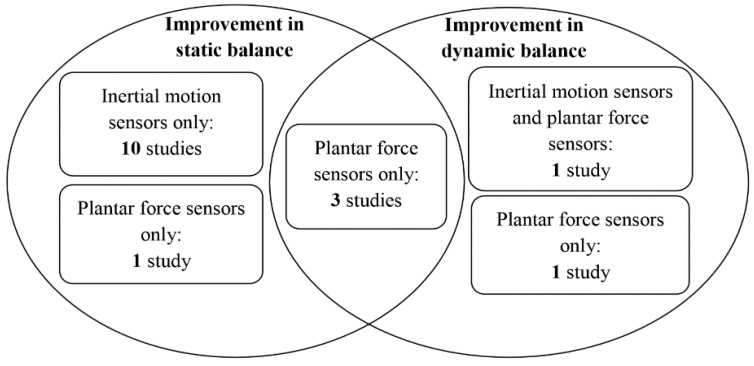
Overview of effectiveness of the devices and type of sensors.

There was one study reporting only four out of 10 subjects showed balance improvements upon using the biofeedback system integrated with inertial motion sensors [68]. However, all the remaining 16 studies concluded that biofeedback information based on the measurements of wearable sensors enhanced either static or dynamic balance, or both of them immediately or in longer follow-up time period significantly (Table 9). An overview of the effectiveness of the devices on static and dynamic balance is summarized in Figure 2. There is a general trend that inertial motion sensors were able to enhance static balance, while plantar force sensors were able to enhance dynamic balance (Figure 2). Three studies were considered to be with high level of evidence (individual randomized controlled trials with narrow confidence intervals), and with recommendation of grade A (strong recommendation that is expected to be followed, unless there are compelling reasons to deviate from the recommendation in an individual) [43,47,50]. These studies revealed that that implementing force sensors and inertial motion sensors together could enhance dynamic balance significantly in patients with stroke or Parkinson’s disease [47]; while inertial motion sensors only could significantly enhance static balance only in patients with diabetes [43], and plantar force sensors only could significantly enhance static and dynamic balance in patients with stroke [50].

#### 4.9.1. Effect of Inertial Motion Sensors on Static Balance

A total of eleven reviewed studies evaluated the effects of the use of inertial motion sensors on static balance. Among them, except one study found positive effects in only four out of 10 patients with bilateral vestibular issues [68], while all ten the other studies reported that the devices with inertial motion sensors significantly improve static balance performance. Inertial motion sensors (accelerometers, gyroscopes, and magnetometers) were attached to the head and the trunk to measure the head and trunk tilts, respectively. To measure the 3D linear and angular trunk kinematics, the inertial motion sensors were placed at the level of center of mass at the posterior lower back. A certain threshold for allowable head/trunk sway was pre-defined for each subject. Instant visual [67,75], auditory [43,64,65,89], vibrotactile [32,36,68,110], and electrotactile [63] biofeedback information were delivered to the users when the postural sway, as measured by the inertial motion sensors, exceeded the thresholds. No biofeedback would be generated if the angle of head/trunk tilt was less than the threshold. Healthy young [32,75,89,110] and older adults [64,65], and patients with spinal cord injury [63], Parkinson’s disease [36,67], and type 2 diabetic peripheral neuropathy [43] were recruited and significant reduction in postural sway in mediolateral and/or anteroposterior directions during standing with the use of the devices were revealed [89].

#### 4.9.2. Effect of Inertial Motion Sensors on Dynamic Balance

None of the involved study aimed to enhance dynamic balance with inertial motion sensors only.

#### 4.9.3. Effect of Inertial Motion Sensors and Plantar Force Sensors on Dynamic Balance

One study tested if a visual biofeedback system with both plantar force sensors and inertial motion sensors enhanced dynamic balance of suhjects with balance and gait disorders [47]. A pair of smart shoes was fabricated by attaching four force sensors to toe, the second and fifth metatarsal heads, and heel at left and right sides seperately. The ground reaction force detected by the force sensors was used to quantitatively analyse the onset and duration of stance/swing phrases and weight bearing at the affected and sound limbs during walking. Additionally, seven inertial measurement units were mounted at the lower back, thigh, shank and foot to capture the step lengths and angles of hips, knees, and ankles at both sides during walking. Each measurement unit consisted of a tri-axial accelerometer, a magnetometer, and a gyroscope. The plantar force data, joint angles, step lengths, stride widths, and toe-out angles of the affected and sound limbs were displayed on the screen of a tablet computer wirelessly for the visual feedback, with a sampling rate of 30 Hz. If these parameters were found to be asymmetrical between affected and sound limbs, visual information would be provided to the patients to adjust their lower-limb joint angles, speed, step length, and/or weight bearing during walking. Patients with Parkinson’s disease and stroke were recruited in this study. All subjects revealed significant improvement of balance during walking with this device.

#### 4.9.4. Effect of Plantar Force Sensors on Static Balance

One study evaluated the static balance effects of using a vibroactile biofeedback system with force sensors put at the plantar surface of the foot [28]. Four vibrators were put at the anterior, posterior, left and right sides of upper trunk to indicate the anterioposterior and mediolateral postural sway, respectively. The force sensors were put at the plantar surface of first and fifth metatarsal heads and heel at the left and right feet. The measured forces of each sensor during three quite standing trials were recorded and averaged. The averaged values were then multipled by 110% to set the threshold for each sensor for allowable postural sway. Once the measured force exceeded this threshold during quite standing, the corresponding vibrator would be actived to remind the user to move to the opposite direction of the vibrator. Healthy young and older subjects participated in this study. They revealed significant reduction of COP sway during quiet standing with eyes-closed with this device.

#### 4.9.5. Effect of Plantar Force Sensors on Dynamic Balance

A total of four studies evaluated the dynamic balance effects of biofeedback systems with plantar force sensors. The force sensors were put at the heel and forefoot to detect the onset of heel-strike, flat foot and toe-off, and stance time during walking. Clues about the occurrence of heel strike and toe-off, as detected by force sensors, were provided to users via auditory [50], visual-auditory [51], or vibrotactile [48,49] biofeedback information. The magnitude of biofeedback information was proportional to the asymmetry ratio of stance/swing time and weight bearing between left and right limbs. Lower-limb amputees [51], patients with stroke [49,50], and healthy young adults [48] were recruited. They revealed significant improvements of dynamic balance and gait symmetry during walking upon using the devices.

## 5. Discussion

This investigation of the literatures about biofeedback systems incorporated with wearable sensors demonstrates the potential of applying wearable sensors and biofeedback to enhance static and dynamic balance performance in patients and aged population. Furthermore, this systematic review also builds on the previous research by examining specific design of biofeedback devices that may influence the efficacy of improving balance.

### 5.1. Effectiveness

Overall, evidence supports the effectiveness of biofeedback systems in enhancing static and dynamic balance among healthy adults and patients with balance disorders. The three studies with high level of evidence and high level of recommendation strongly supported the effectiveness of inertial motion sensors and force sensors in enhancing static and dynamic balance [43,47,50]. The subjects in these three studies included patients with stroke, Parkinson’s disease, and diabetes. One study reported only 4 out of 10 patients with severe bilateral vestibular loss showed balance improvements upon using the biofeedback system integrated with inertial motion sensors [68]. The remaining thirteen studies supported the effectiveness of inertial motion sensors and force sensors in enhancing static and dynamic balance. The subjects in these studies included lower limb amputees, patients with stroke, Parkinson’s disease, and diabetes, and healthy young and older adults.

The wearable characteristics of the sensors would enable them to provide augmented feedback in indoor and outdoor conditions. Significantly reduced postural sway, weight bearing asymmetry, and gait variability were achieved upon using them. Wearable sensors enable objective assessment of balance and gait abnormalities compared with assessments based on clinician/researcher’s subjective perception [47,112,113,114]. It has been recommended that some wearable computerized devices could assist clinical doctors and therapists to further understand the balance conditions of the patients [47]. Further optimizations of the devices included in this review would provide opportunities to achieve this extensively in the near future.

### 5.2. Wearable Sensors in Static and Dynamic Conditions

The wearable sensors that appeared in the publications reviewed in this paper could be divided into two categories: (1) inertial motion sensors, including accelerometers, gyroscopes, and magnetometers; and (2) plantar force/pressure sensors. Information about the body tilt, joint coordination, weight bearing asymmetry, plantar force distribution, and gait variability were detected based on these sensors.

It can be seen that inertial motion sensors were applied mainly in detecting body sway at static conditions. When it comes to dynamic situations, plantar force sensors were needed [47]. One major reason is that while inertial motion sensors could detect trunk and head movements [32,36,43,63,64,65,67,68,75,89,110], they could not easily reflect the conditions of each of the leg during walking. Meanwhile, plantar force sensors could provide temporal and kinetic information of each of the two legs. Step-to-step variability and symmetry between the two legs can be assessed, which have been found to be associated with dynamic balance control during walking [48,50,51]. These strategies are helpful to improve balance by compensating declined plantar sensation in amputees [51], or patients with neurological problems [47,49,50].

### 5.3. Biofeedback Information

While some previous studies have suggested that wearable sensors could monitor the body motion outside laboratory environment accurately and reliably [44,45,48,69], addition of real-time biofeedback could have the potential effect of improving balance. Currently, single or multiple biofeedback information were provided, including visual [43,47,51,67,75,110], auditory [43,50,64,65,89], vibrotactile [28,32,36,48,49,68,110], and electrotactile [51,63,111]. Comparing with showing visual biofeedback information on a large screen [43,51,67,75,110], delivering the visual information through a smaller and portable display [47] or the auditory biofeedback through a pair of earphones makes the devices more portable. The electrotactile and vibrotactile feedbacks further promote this design by providing electrotactile or vibrotactile stimulations to the surface of skin. It has been suggested that the tactile feedbacks do not hinder daily tasks of speaking, eating, seeing and hearing, since the tactile stimulations are received at users’ skin [68,115]. With the non-invasive stimulations and easy-to-operate settings, the user’s acceptance of these devices recorded by verbal-reporting was suggested to be excellent, which imply that such devices could potentially be used as balance and gait control aid in the future [45,48].

### 5.4. Future Directions

This review highlights several issues that merit further exploration. Firstly, most early studies only focused on enhancing static balance or postural stability during standing [28,32,36,43,63,64,65,66,67,68,75,89,110,111]. Some recent research attempted to enhance dynamic balance, but the tasks were simple and limited to achieve symmetric weight bearing and stance/swing time by detecting heel strike and toe-off in gait only [47,48,49,50,51]. Future attempts should focus on enhancing dynamic balance and gait control, which is more complex and common in daily life.

Secondly, although the existing research provides evidence for the ability to improve balance performance by providing biofeedback based on body motion measured by wearable sensors, it is not clear what effects of these improvements have on function in daily life or risk of falls. Outcome measurements could be expanded to include functional measures of balance performance relevant to daily life, including ascending and descending stairs, walking on slopes, or some dual-task conditions. Future studies should also incorporate the prospective long-term effect of these devices on simple and more functional balance assessments.

Thirdly, the decision-making of choosing appropriate sensors could be made after thorough evaluations and be further utilized based on the user’s condition in the future. Combined inertial motion and force sensors should be superior for the development of new wearable device, as they could compensate each other’s function [47]. There are various tailor-made options for patients with different types of sensory deficiency. The inertial motion sensors were shown to be able to detect the movement of the whole body and the body segments accurately in healthy older adults and patients with balance and gait disorders before [44,45,48,69]. However, the inertial motion sensors could not measure the foot-floor contact surface information, which could be eased by force sensors put at the plantar surface of the foot. Plantar tactile sensation plays an important role in balance control [116,117,118], as it provides instantaneous and continuous information about support surface characteristics [119] and the body’s relative movement to the foot [120] to the central nervous systems. Declined plantar tactile sensitivity can induce poor balance and predispose risk of falls [121]. Aging, diabetic neuropathy, Parkinson’s disease, and rheumatoid arthritis can lead to impairments in plantar tactile sensation [122]. The force sensors could help those people by providing them additional foot-floor contact information, which compensates the pathological plantar sensory deficits. Furthermore, the force sensors put at the left and right foot could also help distinguish the plantar force distribution of the affected and sound sides. This makes the plantar force sensors a suitable option for patients with stroke, as well as amputees [123], who commonly have different conditions regarding the sound and affected sides [124].

Fourthly, the biofeedback devices could be developed more wearable and more appropriate for outdoor uses in the future. Some of the previous biofeedback devices were connected physically to computers for analyzing signals and sending feedback [34,37,39,40,41]. These devices acted as indoor/laboratory-based balance training devices only. With current state-of-the-art smartphone and other smart product applications, advancement of wearable sensors and Bluetooth connections, the devices could be developed more lightweight, with more powerful calculation capacity and smaller size in different wearable products (shoes, apparels or accessories) in the future. The inertial motion sensors could be incorporated into a single package and be attached to the belt. The thin-film force sensors could be inserted into insoles and the relevant electronics be put at shoe soles to develop some kinds of smart shoes. The inertial motion sensors and software in a smartphone could also be utilized and be developed as potential mobile balance aids for daily uses. With larger number of force and motion sensors been used, it would be more feasible to measure the movement trajectory of center of pressure during standing and walking [26,27], which is an important indicator of balance performance and falling risk [81,85]. Visual, auditory, and tactile biofeedback information could be used as reminder during laboratory-based as well as home-based rehabilitation training sessions. When considering the requirements of outdoor training and daily balance aid, the choice of tactile biofeedback might be more appropriate, as it does not hinder daily tasks of speaking, eating, seeing and hearing [68,115]. The tactile biofeedback information could be delivered to human body wirelessly. In addition, previous devices detected COM position only using inertial motion sensors, but the XcoM appears to be more related to dynamic balance performance [91,92]. Considerations could be given to measure the displacement of XcoM by using inertial motion sensors and force sensors together in the future, as they could measure the movements of both COM and COP, which could be used to calculate the movements of XcoM. All these possible design characteristics provide these devices the potential to act as balance aids in daily life, as well as the home-based rehabilitation training devices that could be used anytime and at anywhere, especially when some of the wearable balance-improving devices have been suggested to be as effective as therapist’s verbal instructions [47].

Fifthly, most of the wearable devices have not been fully utilized for balance enhancement in commercial market yet. Attempts have been made to turn commercially available smartphones into wearable balance-improving biofeedback devices. Researchers used built-in inertial motion sensors to monitor postural balance [72,89], and provided auditory feedback from the speakers of the smartphone to remind on the necessary changes in postures [89]. The possible reasons for these devices not being commercialized yet might be that research in this area have been limited causing failure to catch the attention of the industry. The effects of these devices on multiple representative subject samples are needed to ascertain if these devices are widely sufficient in different user groups. The effects of these wearable devices on various dynamic conditions have not been fully understood yet, since most previous studies investigated static balance evaluation only. Since these devices have not been widely applied, comments from the physicians were lacked. Accumulating evaluation data and user’s feedback is necessary to optimize the functionality of the wearable balance training system for people with various degrees of balance impairments and age.

Finally, in order to strengthen the evidence base of enhancing static and dynamic balance performance through wearable biofeedback systems, future studies could recruit larger, more representative samples, and apply a standard set of assessment methods to allow possible cross-study comparison and meta-analysis. Randomized controlled trials should also be conducted.

### 5.5. Limitations

Seventeen clinical trials evaluated the effectiveness of biofeedback systems based on wearable sensor measurements in a total of 282 subjects were reviewed. Due to heterogeneous outcome measures, the quantitative synthesis and meta-analysis were excluded unfortunately. Furthermore, the quality of evidence in this review was mixed, with a risk of bias, as some studies did not adopt randomization.

Studies incorporated a variety of experimental protocols and different outcomes to assess the effectiveness of the devices. This led to the difficulty of identifying the optimal device design and providing specific recommendations. The lack of long-term follow-up period also made it difficult to determine the existence of retained effect of these devices.

## 6. Conclusions

A synthesis of research examining the effect of biofeedback on static and dynamic balance performance based on body motion information measured by wearable sensors suggests that most of these devices are effective. Inertial motion sensors and plantar force sensors have been adopted to capture the body motion in static and dynamic conditions, including head/trunk tilt, lower-limb joint co-ordination, weight bearing asymmetry, and spatial-temporal gait variability. A variety of feedbacks (visual, auditory, vibrotactile and electrotactile) were delivered to the users. The design of these devices could be further optimized by applying some state-of-the-art technologies to make the devices more lightweight, with more powerful processing capacities, smaller size, and higher usability. Some smart products could be integrated/connected with wearable sensors wirelessly to compute body balance and provide various biofeedback information. These devices have a good potential to be used as laboratory- and home-based rehabilitation training devices, as well as balance aids in daily life. Numerous various populations could be benefit from these devices in the future.

## Figures and Tables

**Table 1:** Overview of the sensing mechanism of wearable sensors.

Type of Wearable Sensor		Outcome Measurement	Location of Sensor
Inertial motion sensor	Accelerometer	Linear acceleration of X, Y, and Z movements in a three dimensional space	Body segment
	Gyroscope	Angular velocity: extent and rate of rotation in a three dimensional space (roll, pitch, and yaw)	Body segment
	Magnetometer	Direction: absolute angular movements relative to the Earth’s magnetic field	Body segment
Planter force sensor		Plantar force/pressure information	Plantar surface of foot

**Table 2:** Levels of Evidence (Oxford Centre for Evidence-based Medicine—March 2009) [107].

Level	Therapy/Prevention, Aetiology/Harm
**1a**	Systematic Review (with homogeneity) of Randomized Controlled Trials
**1b**	Individual Randomized Controlled Trial (with narrow Confidence Interval)
**1c**	All or none
**2a**	Systematic Review (with homogeneity) of cohort studies
**2b**	Individual cohort study (including low quality Randomized Controlled Trial; e.g., <80% follow-up)
**2c**	“Outcomes” Research; Ecological studies
**3a**	Systematic Review (with homogeneity) of case-control studies
**3b**	Individual Case-Control Study
**4**	Case-series (and poor quality cohort and case-control studies)
**5**	Expert opinion without explicit critical appraisal, or based on physiology, bench research or “first principles”

**Table 3:** Grades of Recommendation (Oxford Centre for Evidence-based Medicine—March 2009) [107].

Grade	Contents
**A**	consistent level 1 studies
**B**	consistent level 2 or 3 studies or extrapolations from level 1 studies
**C**	level 4 studies or extrapolations from level 2 or 3 studies
**D**	level 5 evidence or troublingly inconsistent or inconclusive studies of any level

**Table 4:** The Downs and Black Quality List [108].

Subscale	Item	Index	Score					
			5	4	3	2	1	0
**Reporting**	**1**	Is the hypothesis/aim/objective of the study clearly described?	-	-	-	-	Y	N
	**2**	Are the main outcomes to be measured clearly described in the Introduction or Methods section?	-	-	-	-	Y	N
	**3**	Are the characteristics of the patients included in the study clearly described?	-	-	-	-	Y	N
	**4**	Are the interventions of interest clearly described?	-	-	-	-	Y	N
	**5**	Are the distributions of principal confounders in each group of subjects to be compared clearly described?	-	-	-	Y	P	N
	**6**	Are the main findings of the study clearly described?	-	-	-	-	Y	N
	**7**	Does the study provide estimates of the random variability in the data for the main outcomes?	-	-	-	-	Y	N
	**8**	Have all important adverse events that may be a consequence of the intervention been reported?	-	-	-	-	Y	N
	**9**	Have the characteristics of patients lost to follow-up been described?	-	-	-	-	Y	N
	**10**	Have actual probability values been reported (e.g., 0.035 rather than <0.05) for the main outcomes except where the probability value is less than 0.001?	-	-	-	-	Y	N
**External Validity**	**11**	Were the subjects asked to participate in the study representative of the entire population from which they were recruited?	-	-	-	-	Y	N/UD
	**12**	Were those subjects who were prepared to participate representative of the entire population from which they were recruited?	-	-	-	-	Y	N/UD
	**13**	Were the staff, places, and facilities where the patients were treated, representative of the treatment the majority of patients receive?	-	-	-	-	Y	N/UD
**Internal Validity-Bias**	**14**	Was an attempt made to blind study subjects to the intervention they have received?	-	-	-	-	Y	N/UD
	**15**	Was an attempt made to blind those measuring the main outcomes of the intervention?	-	-	-	-	Y	N/UD
	**16**	If any of the results of the study were based on “data dredging”, was this made clear?	-	-	-	-	Y	N/UD
	**17**	In trials and cohort studies, do the analyses adjust for different lengths of follow-up of patients, or in case-control studies, is the time period between the intervention and outcome the same for cases and controls?	-	-	-	-	Y	N/UD
	**18**	Were the statistical tests used to assess the main outcomes appropriate?	-	-	-	-	Y	N/UD
	**19**	Was compliance with the intervention/s reliable?	-	-	-	-	Y	N/UD
	**20**	Were the main outcome measures used accurate (valid and reliable)?	-	-	-	-	Y	N/UD
**Internal Validity-Confounding (Selection Bias)**	**21**	Were the patients in different intervention groups (trials and cohort studies) or were the cases and controls (case-control studies) recruited from the same population?	-	-	-	-	Y	N/UD
	**22**	Were study subjects in different intervention groups (trials and cohort studies) or were the cases and controls (case-control studies) recruited over the same period of time?	-	-	-	-	Y	N/UD
	**23**	Were study subjects randomised to intervention groups?	-	-	-	-	Y	N/UD
	**24**	Was the randomised intervention assignment concealed from both patients and health care staff until recruitment was complete and irrevocable?	-	-	-	-	Y	N/UD
	**25**	Was there adequate adjustment for confounding in the analyses from which the main findings were drawn?	-	-	-	-	Y	N/UD
	**26**	Were losses of patients to follow-up taken into account?	-	-	-	-	Y	N/UD
**Power**	**27**	Did the study have sufficient power to detect a clinically important effect where the probability value for a difference being due to chance is less than 5%?	Size of smallest intervention group					
			> *n*_8_	*n*_7_ − *n*_8_	*n*_5_ − *n*_6_	*n*_3_ − *n*_4_	*n*_1_ − *n*_2_	< *n*_1_

-Note: Y: yes; P: partially; N: no; UD: unable to determine.

**Table 5:** Level of evidence and grade of recommendation (*n* = 17).

Study	Level of Evidence	Design	Level of Recommendation
Afzal *et al.* 2015 [49]	3B	Individual Case-Control Study	B
Byl *et al.* 2015 [47]	1B	Individual Randomized Controlled Trial	A
Crea *et al.* 2015 [48]	3B	Individual Case-Control Study	B
Grewal *et al.* 2015 [43]	1B	Individual Randomized Controlled Trial	A
Ma *et al.* 2015 [28]	3B	Individual Case-Control Study	B
Caudron *et al.* 2014 [67]	3B	Individual Case-Control Study	B
Halicka *et al.* 2014 [75]	3B	Individual Case-Control Study	B
Franco *et al.* 2013 [89]	3B	Individual Case-Control Study	B
Nanhoe-Mahabier *et al.* 2012 [36]	3B	Individual Case-Control Study	B
Nataraj *et al.* 2012 [63]	3B	Individual Case-Control Study	B
Sungkarat *et al.* 2011 [50]	1B	Individual Randomized Controlled Trial	A
Alahakone *et al.* 2010 [110]	3B	Individual Case-Control Study	B
Janssen *et al.* 2010 [68]	3B	Individual Case-Control Study	B
Giansanti *et al.* 2009 [64]	3B	Individual Case-Control Study	B
Lee *et al.* 2007 [51]	3B	Individual Case-Control Study	B
Chiari *et al.* 2005 [65]	3B	Individual Case-Control Study	B
Wall *et al.* 2001 [32]	3B	Individual Case-Control Study	B

**Table 6:** Assessing results of the Downs and Black Quality List (*n* = 17).

Study	Score of Subscale and Index																											
	Reporting										External Validity			Internal Validity-Bias							Internal Validity-Confounding (Selection Bias)						Power	Total
	1	2	3	4	5	6	7	8	9	10	11	12	13	14	15	16	17	18	19	20	21	22	23	24	25	26	27	
Afzal *et al.* 2015 [49]	1	1	1	1	0	1	1	0	1	1	0	0	1	0	0	1	1	1	1	1	1	0	0	0	1	1	0	17
Byl *et al.* 2015 [47]	1	1	1	1	1	1	1	0	1	0	0	1	0	0	0	1	1	1	1	1	1	0	1	1	1	1	0	19
Crea *et al.* 2015 [48]	1	1	1	1	0	1	1	0	1	0	0	0	0	0	0	1	1	1	1	1	1	0	0	0	1	1	0	15
Grewal *et al.* 2015 [43]	1	1	1	1	1	1	1	1	1	1	0	1	1	1	0	1	1	1	1	1	1	1	1	1	1	1	1	25
Ma *et al.* 2015 [28]	1	1	1	1	1	1	1	0	1	1	0	0	0	0	0	1	1	1	1	1	1	0	1	0	1	1	1	19
Caudron *et al.* 2014 [67]	1	1	1	1	1	1	1	0	1	0	0	0	1	0	0	1	1	1	1	1	1	0	0	0	1	1	0	17
Halicka *et al.* 2014 [75]	1	1	1	1	1	1	1	0	1	1	0	0	0	0	0	1	1	1	1	1	1	0	0	0	1	1	0	17
Franco *et al.* 2013 [89]	1	1	1	1	1	1	1	0	1	0	0	0	0	1	0	1	1	1	1	1	1	0	1	0	1	1	0	18
Nanhoe-Mahabier *et al.* 2012 [36]	1	1	1	1	1	1	1	1	1	1	0	0	1	0	0	1	1	1	1	1	1	0	1	0	1	1	0	20
Nataraj *et al.* 2012 [63]	1	1	1	1	1	1	0	0	1	0	0	0	1	0	0	1	1	0	1	1	1	1	0	0	1	0	0	15
Sungkarat *et al.* 2011 [50]	1	1	1	1	1	1	1	1	1	1	0	1	1	0	0	1	1	1	1	1	1	1	1	1	1	1	0	23
Alahakone *et al.* 2010 [110]	1	1	1	1	0	1	1	0	1	0	0	0	0	0	0	1	1	1	1	1	0	1	0	0	1	1	0	15
Janssen *et al.* 2010 [68]	1	1	1	1	1	1	0	0	1	1	0	0	0	0	0	1	1	1	1	1	1	0	1	0	1	1	0	17
Giansanti *et al.* 2009 [64]	1	1	1	1	0	1	1	0	1	1	0	0	0	0	0	1	1	1	1	1	1	0	0	0	1	1	0	16
Lee *et al.* 2007 [51]	1	1	1	1	1	1	1	0	1	1	0	0	1	0	0	1	1	1	1	1	1	0	0	0	1	1	0	18
Chiari *et al.* 2005 [65]	1	1	1	1	0	1	1	0	1	0	0	0	0	0	0	1	1	1	1	1	1	0	0	0	1	1	0	15
Wall *et al.* 2001 [32]	1	1	1	1	0	1	1	0	1	0	0	0	0	0	0	1	1	1	1	1	1	0	0	0	1	1	0	15

**Table 7:** Subject characteristics (*n* = 17).

Study	Sample Size (Gender, F/M)	Group (Sample Size, n): Mean (SD) Age, Years	Sample Characteristics		
			Physical	Cognitive	Fall History
Afzal *et al.* 2015 [49]	9 (1/8)	Intervention (9):	-Stroke: clear symptoms of lower-limb weakness at the paretic side. -Young healthy: no history of musculoskeletal or neurological disorders.	Not specified	Not specified
		-Stroke (4): 64.8 (9.5)			
		-Healthy (5): 26.2 (3.3)			
Byl *et al.* 2015 [47]	24 (8/16)	Intervention (12):	-With gait impairments and one year or more post stroke or diagnosis of PD. -Independent in self-care, could rise from a chair, and walk without personal assistance for a minimum of 100 feet. -PD: scored I to III on the Hoehn and Yahr Scale -Stroke: a minimum score of 10 of the lower-limb evaluated by the Cafe 40-Functional Independence Scale	-Able to follow instructions -No severe depression.	Not specified
		-PD (7): 68.5 (3.6)			
		-Stroke (5): 66.2 (5.0)			
		Control (12):			
		-PD (5): 70 (2.9)			
		-Stroke (7): 60.8 (5.4)			
Crea *et al.* 2015 [48]	10 (6/4)	Intervention (10): 27.0 (1.8)	-Able-bodied	Not specified	Not specified
Grewal *et al.* 2015 [43]	39 (20/19)	Intervention (19): 62.6 (8.0)	-Type 2 diabetes with peripheral neuropathy; able to independently walk for 2m. -No vestibular or central neurological dysfunction, musculoskeletal abnormality, active foot ulcers, Charcot’s joints or a history of balance disorder unrelated to DPN.	No cognitive dysfunction	Not specified
		Control (20): 64.9 (8.5)			
Ma *et al.* 2015 [28]	30 (13/17)	Intervention (30):	-Fully independent, living in a community-based setting, and were capable of ambulation without assistive devices. -No neurological or vestibular disorders, diabetes, severe cardiovascular or pulmonary diseases, previous history of foot injury, foot deformity, amputation of the lower limbs, inability to attend the necessary re-evaluations, or inability to follow the instructions and procedures	Able to follow instructions	Not specified
		-Elderly (15): 70.1 (3.7)			
		-Young (15): 26.7 (2.9)			
Caudron *et al.* 2014 [67]	17 (7/10)	Intervention (17): 61.9 (8.2)	-Patients with idiopathic PD. -Scored II to III on the Hoehn and Yahr Scale and UPDRS motor score	Not specified	With or without
Halicka *et al.* 2014 [75]	20 (11/9)	Intervention (20): 22.6 (nil)	Healthy young subjects did not report any neurological, orthopaedic, or balance impairments.	Not specified	Not specified
Franco *et al.* 2013 [89]	20 (11/9)	Intervention (20): 26.5 (3.7)	Healthy young subjects with no history of sensory or motor problems, neurological diseases, or disorders.	Not specified	Not specified
Nanhoe-Mahabier *et al.* 2012 [36]	20 (4/16)	Intervention (10): 59.3 (2.0)	-Patients with PD. -With no causes of balance impairment other than PD, able to walk without walking aids, and no severe co-morbidity.	No cognitive dysfunction	Not specified
		Control (10): 58.6 (2.5)			
Nataraj *et al.* 2012 [63]	1 (1/0)	Intervention (1): nil (nil)	Patient with thoracic-4 level complete paraplegia.	Not specified	Not specified
Sungkarat *et al.* 2011 [50]	35 (11/24)	Intervention (17): 52.1 (7.2)	-Patients with first episode of unilateral stroke with hemiparesis; orpington prognostic score at initial assessment between 3.2 and 5.2 (moderately severe); able to walk at least 10 m with or without assistance; stable medical condition; and to participate. -Patients without any comorbidity or complication that would preclude gait training, severe leg spasticity (Modified Ashworth Scale ≥3 [50]), neglect, or missed more than three training sessions.	No impaired cognition and/or communication	Not specified
		Control (18): 53.8 (11.2)			
Alahakone *et al.* 2010 [110]	6 (3/3)	Intervention (6): 23.2 (nil)	Healthy young subjects	Not specified	Not specified
Janssen *et al.* 2010 [68]	20 (8/12)	Intervention (10): 63.1 (9.3)	Patients with severe bilateral vestibular losses (flexia or hyporeflexia). With severe balance problems	Not specified	>5 times falls per year
		Control (10): 40-65			
Giansanti *et al.* 2009 [64]	9 (nil)	Intervention (9): 55.0 (33-71)	Healthy subjects	Not specified	Not specified
Lee *et al.* 2007 [51]	7 (2/5)	Intervention (7): 38.9 (14.1)	Lower-limb amputees with no orthopaedic or neurological conditions, disabling arthritis, uncorrected visual problems, dizziness or vertigo, use of assistive walking devices, joint injury, or joint implants	Not specified	Not specified
Chiari *et al.* 2005 [65]	9 (nil)	Intervention (9): 55.0 (33-71)	Healthy subjects	Not specified	Not specified
Wall *et al.* 2001 [32]	6 (4/2)	Intervention (6): 24.8 (22-29)	Healthy subjects	Not specified	Not specified

**Table 8:** Device characteristics (*n* = 17).

Study	Type of Sensors	Location of Sensors	Type of Biofeedback	Function of Device
Afzal *et al.* 2015 [49]	Plantar force sensors	Heel, toe, 1st and 5th MT heads	Vibrotactile	Diagnose gait abnormalities and provide vibration feedback to help compensate for the asymmetric gait.
Byl *et al.* 2015 [47]	Plantar force sensors	Toe, 1st and 2nd MTP, 4th and 5th MTP, and heel	Visual	Gait training with visual kinematic feedback on iPad.
	Accelerometer	Shank and thigh		
	Magnetometer			
	Gyroscope			
Crea *et al.* 2015 [48]	Plantar force sensors (64 at each insole)	Plantar surface of the foot	Vibrotactile	Provide simultaneous vibration based on the detected gait phase transitions.
Grewal *et al.* 2015 [43]	Accelerometer	Shank, thigh and lower back	Auditory & Visual	Provide audio-visual feedback on a display of the sway of COM and ankle joints
	Magnetometer			
	Gyroscope			
Ma *et al.* 2015 [28]	Plantar force sensors	Heel, 1st and 5th MT heads	Vibrotactile	Provide vibrotactile feedback of postural sway.
Caudron *et al.* 2014 [67]	Accelerometer	Cranial vertex and the spine processes of the T7-T8	Visual	Real time biofeedback of anterior-posterior trunk and head tilts
	Magnetometer			
Halicka *et al.* 2014 [75]	Accelerometer	Posterior side of T4, L5	Visual	Capture body sway and provide visual biofeedback
	Force plate			
Franco *et al.* 2013 [89]	Accelerometer	Posterior side of L5	Auditory	Monitor the trunk angular evolution during bipedal stance and improve user’s balance through auditory biofeedback by earphone
	Magnetometer			
	Gyroscope of a smartphone			
Nanhoe-Mahabier *et al.* 2012 [36]	Angular velocity sensors	Lower back at level L1-L3	Vibrotactile	Deliver vibrotactile feedback of trunk sway to head
Nataraj *et al.* 2012 [63]	Accelerometer	Pelvis and torso	Electrotactile	Estimate COM acceleration using inputs from body-mounted accelerometer measurements.
				Deliver stimulation via surgically implanted intramuscular electrodes to bilateral muscle groups of trunk and lower limb
Sungkarat *et al.* 2011 [50]	Plantar force sensors	Heel of the paretic foot	Auditory	Rehabilitation and gait training based on footswitch and the amount of weight bearing at the paretic limb
Alahakone *et al.* 2010 [110]	Accelerometer	Lower back	Vibrotactile & Visual	Measure the ML trunk tilt angles
	Gyroscope			Custom-developed software for data processing, data display and feedback generation
	Temperature sensor			
Janssen *et al.* 2010 [68]	Accelerometer	Head or upper trunk	Vibrotactile	Detect head or body tilt
				Deliver vibrotactile biofeedback to the waist.
Giansanti *et al.* 2009 [64]	Accelerometer	Body centre of mass (COM).	Auditory	Assess the trunk sway and provide biofeedback information
	Gyroscope			
Lee *et al.* 2007 [51]	Plantar force sensors	Heel and the 3rd MT head of the prosthetic foot	Electrical & Visual-auditory	Detect heel strike and toe off.
				Provide sub-threshold low-level electrical stimulation to the quadriceps, and visual-auditory biofeedback on a screen
Chiari *et al.* 2005 [65]	Accelerometer	Trunk	Auditory	Measure the linear accelerations of the trunk in anteroposterior and mediolateral directions
				Provide audio-biofeedback via headphones
Wall *et al.* 2001 [32]	Accelerometer	Head	Vibrotactile	Measure lateral head tilt and mount vibrotactile elements on the body to display head tilt
	Gyroscope			

**Table 9:** Study outcome characteristics (*n* = 17).

Study	Assessment Point	Outcome Measures	Measurement Tool	Results	Balance Improvement
Afzal *et al.* 2015 [49]	(1) Pre-test	-Postural stability during standing; -Gait asymmetry	Smartphone with inertial sensors	(1) Vibration cue based on temporal information was more effective than intensity information	Yes, static and dynamic balance
	(2) Post-test			(2) Individuals with stroke revealed significant improvement in gait symmetry with minimal disturbance caused to the balance and gait speed as an effect of the biofeedback.	
Byl *et al.* 2015 [47]	(1) Pre-test	-Mobility (gait speed, step length, endurance, and quality) -Balance (Berg Balance) -Range of motion and strength of joints.	-Force sensors -Inertial motion sensors -Clinical tests	(1) All subjects revealed significant gains in mobility, balance, range of motion and strength.	Yes, dynamic balance
	(2) Post-test			(2) Subjects with chronic post stroke achieved greater strength gains on the affected side than subjects with PD.	
	(3) 6 weeks			(3) Dynamic visual kinematic feedback from wireless pressure and motion sensors had similar positive effects as verbal and therapist feedback.	
Crea *et al.* 2015 [48]	(1) Pre-test	-No. of correct/wrong detection of gait phrases -Temporal gait phrases	-Questionnaire -Force sensors	(1) High recognition of feedback information	Yes, dynamic balance
	(2) Post-test			(2) Time-discrete low-intensity feedback was readily perceived by humans and potentially can assist gait control	
Grewal *et al.* 2015 [43]	(1) Pre-test	Postural stability during standing	Inertial motion sensors	(1) Significant reduction in COM sway after training.	Yes, static balance
				(2) A higher postural stability deficit (high body sway) at baseline was associated with higher training gains in postural balance (reduction in COM sway).	
	(2) Post-test			(3) Significant improvement in postural coordination between the ankle and hip joints.	
Ma *et al.* 2015 [28]	(1) Pre-test	Postural stability during standing;	Force plate	Significant reduction in COP sway after training.	Yes, static balance
	(2) Post-test				
Caudron *et al.* 2014 [67]	(1) Pre-test	-Postural stability -Postural orientation during standing	Motion capture system using reflective markers	Visual biofeedback improved PD patients’ postural orientation and postural stability	Yes, static balance
	(2) Post-test				
Halicka *et al.* 2014 [75]	(1) Pre-test	-COP -Postural stability during standing	-Force plate -Inertial motion sensors	(1) Reduction of body sway was the most significant in the body segment upon receiving the visual biofeedback.	Yes, static balance
	(2) Post-test			(2) The COP position and L5 position provided the best signals for visual biofeedback.	
Franco *et al.* 2013 [89]	(1) Pre-test	Postural stability during standing	Inertial motion sensors	Young healthy individuals were able to efficiently use auditory biofeedback on sagittal trunk tilt to improve their balance in the medial-lateral direction.	Yes, static balance
	(2) Post-test				
Nanhoe-Mahabier *et al.* 2012 [36]	(1) Pre-test	Postural stability during standing	Angular velocity sensors	(1) Patients in the feedback group had a significantly greater reduction in ML and AP postural sway.	Yes, static balance
	(2) Post-test			(2) Greater ML sway angle in controls after training suggested better training effects in the feedback group	
Nataraj *et al.* 2012 [63]	(1) Pre-test	Postural stability during standing	Motion capture system using reflective markers	Compared with constant muscle stimulation employed clinically, controlled stimulations based on COM acceleration improved standing performance more and reduced the upper limb loading required to resist internal postural disturbances by 27%	Yes, static balance
	(2) Post-test				
Sungkarat *et al.* 2011 [50]	(1) Pre-test	Gait speed, step length and single support time asymmetry ratio, balance and amount of load on paretic leg during stance	-Motion capture system -Clinical tests: BBS, TUG	(1) The experimental group demonstrated significant increase in standing and gait symmetry compared with the control group.	Yes, static and dynamic balance
				(2) The experimental group demonstrated three times greater improvement in gait speed than the control group.	
	(2) 3 weeks (60 min × 5 days/week)			(3) Balance improvement was significantly greater for the experimental than the control group	
Alahakone *et al.* 2010 [110]	(1) Pre-test	ML trunk sway during tandem Romberg standing tests	-Inertial motion sensors -Web camera for sighted tests	(1) Feedback was triggered 100% of the time when trunk tilt exceeded the defined threshold.	Yes, static balance
	(2) Post-test			(2) Significant reduction in trunk tilt angle.	
Janssen *et al.* 2010 [68]	(1) Pre-test	Body sway during standing (COP)	Force plate	(1) No significant change in body sway path was observed using biofeedback in six subjects.	Partially yes, static balance
				(2) In four patients, body sway path decreased significantly using biofeedback and sensor on the head in all three activation modes, whereas with sensor on the trunk only one patient showed a significant improvement in sway path in all three activation modes.	
	(2) Post-test			(3) However, the improvement with true biofeedback was only observed in those subjects where an improvement was present in placebo mode as well.	
Giansanti *et al.* 2009 [64]	(1) Pre-test	Changes in angular sway and kinetic energy variables	Inertial motion sensors	Using auditory biofeedback, all subjects significantly reduced pitch, roll and angular velocity with eyes open or closed while standing on a foam surface	Yes, static balance
	(2) Post-test				
Lee *et al.* 2007 [51]	(1) Pre-test	-Single leg quiet standing balance -Dynamic treadmill ambulatory gait performance	Motion capture system	(1) Improvement in balance performance during single leg quiet standing by applying sub-sensory stimulation.	Yes, static and dynamic balance
	(2) Post-test			(2) With visual-auditory biofeedback as a cue for heel contact and toe push-off condition during treadmill ambulation, the dynamic gait performance of amputees was improved.	
Chiari *et al.* 2005 [65]	(1) Pre-test	Postural stability during standing	Force plate	(1) Improved balance upon using the audio-biofeedback system and this improvement was greater when the subject’s balance was challenged by absent or unreliable sensory cues.	Yes, static balance
	(2) Post-test			(2) High correlations were found between the COP displacement and trunk acceleration	
Wall *et al.* 2001 [32]	(1) Pre-test	-Lateral head sway -Postural stability during standing	-Inertial motion sensors -Force plate	Reduced lateral postural sway upon using the head tilt information.	Yes, static balance
	(2) Post-test				

## Abbreviations

The following abbreviations are used in this manuscript:

AP: Anterior-Posterior
BBS: Berg Balance Scale
COM: Center-of-Mass
COP: Center-of-Pressure
DPN: Diabetic Peripheral Neuropathy
IMU: Inertial Measurement Unit
LOS: Limits-of-Stability
MEMS: Micro-Electro-Mechanical Systems
ML: Medial-Lateral
PD: Parkinson’s Disease
PSIS: Posterior Superior Iliac Spine
TUG: Timed Up and Go test
